# Altered Subcellular Localization of a Tobacco Membrane Raft-Associated Remorin Protein by Tobamovirus Infection and Transient Expression of Viral Replication and Movement Proteins

**DOI:** 10.3389/fpls.2018.00619

**Published:** 2018-05-15

**Authors:** Nobumitsu Sasaki, Eita Takashima, Hiroshi Nyunoya

**Affiliations:** ^1^Gene Research Center, Tokyo University of Agriculture and Technology, Fuchu, Japan; ^2^Faculty of Science and Engineering, Waseda University, Tokyo, Japan

**Keywords:** remorin, *Tomato mosaic virus*, plasma membrane, membrane raft, cell-to-cell movement, *Nicotiana benthamiana*, tobacco, BiFC

## Abstract

Remorins are plant specific proteins found in plasma membrane microdomains (termed lipid or membrane rafts) and plasmodesmata. A potato remorin is reported to be involved in negatively regulating potexvirus movement and plasmodesmal permeability. In this study, we isolated cDNAs of tobacco remorins (NtREMs) and examined roles of an NtREM in infection by tomato mosaic virus (ToMV). Subcellular localization analysis using fluorescently tagged NtREM, ToMV, and viral replication and movement proteins (MPs) indicated that virus infection and transient expression of the viral proteins promoted the formation of NtREM aggregates by altering the subcellular distribution of NtREM, which was localized uniformly on the plasma membrane under normal conditions. NtREM aggregates were often observed associated closely with endoplasmic reticulum networks and bodies of the 126K replication and MPs. The bimolecular fluorescence complementation assay indicated that NtREM might interact directly with the MP on the plasma membrane and around plasmodesmata. In addition, transient overexpression of NtREM facilitated ToMV cell-to-cell movement. Based on these results, we discuss possible roles of the tobacco remorin in tobamovirus movement.

## Introduction

Genome replication and intercellular (cell-to-cell) movement of plant viruses depend on various types of host cellular organelles and membrane structures (Heinlein, 2015). Regarding virus-host interactions at molecular levels, *Tobacco mosaic virus* (TMV) and its closely related *Tomato mosaic virus* (ToMV, formerly TMV-L strain), belonging to the genus *Tobamovirus*, are well-studied plant viruses. Both viruses encode at least four proteins: the 126-kDa (126K) and 183-kDa (183K) proteins involved in genome replication, the 30-kDa movement protein (MP) indispensable for movement through plasmodesmata (cytoplasmic channels connecting adjacent cells), and the coat protein (CP) (Heinlein, 2002; Ishikawa and Okada, 2004). Tobamoviruses like TMV and ToMV are thought to replicate the virus genome on the endoplasmic reticulum (ER) and tonoplast membranes and transport it to plasmodesmata through interactions of host factors on ER/actin networks and at ER/microtubule junctions (Ishibashi et al., 2010; Heinlein, 2015). Tobamovirus replication and subsequent movement are proposed to occur in the form of the virus replication complex (VRC), which is composed of viral RNA, the 126K and 183K replication proteins and MP as well as yet undetermined host factors (Liu and Nelson, 2013). CP is required for systemic infection but dispensable for viral replication and intercellular movement (Meshi et al., 1987).

Biochemical and cytological studies on the MP of TMV and ToMV have demonstrated their properties to facilitate intra- and intercellular movement of the virus genome, such as binding to the viral genome (Citovsky et al., 1990), localizing at plasmodesmata (Tomenius et al., 1987), expanding the size exclusion limit of plasmodesmata (Wolf et al., 1989), and associating with the ER and plasma membranes, actin filaments and microtubules (Moore et al., 1992; Heinlein et al., 1995; McLean et al., 1995; Su et al., 2010). The MP is assumed to be involved temporarily in the formation of VRC. During infection, viral RNAs are observed in large cytoplasmic bodies of MPs, which are often regarded and referred to as VRC. Cytoplasmic bodies of the MP in various sizes are formed during viral infection or in cells expressing the protein alone (Heinlein et al., 1998; Liu et al., 2005; Brandner et al., 2008; Sasaki et al., 2009, 2012, 2014). In addition, many recent studies have demonstrated that the 126K protein also plays an essential role in viral intra- and intercellular movement as well as replication (Hirashima and Watanabe, 2001; Tamai and Meshi, 2001; Guenoune-Gelbart et al., 2008). The transient expression of the 126K protein alone triggers the formation of cytoplasmic bodies of the protein, which are similar to VRC observed during virus infection (Heinlein et al., 1998; Más and Beachy, 1999; dos Reis Figueira et al., 2002; Liu et al., 2005; Wang et al., 2010). The temporary association of 126K-containing VRC with the MP after replication may be required for the transition from VRC to the viral RNA-MP complex that transports the viral genome to plasmodesmata. In cells co-expressing the 126K protein and the MP transiently, mobile 126K bodies in the cytoplasmic streaming are observed to stop at the terminal part of filamentous structures of the MP, which are assumed to be associated with microtubules (Heinlein et al., 1998; Más and Beachy, 1999; Ashby et al., 2006; Brandner et al., 2008), and afterward translocate together with the MP as a 126K-MP complex (Sasaki et al., 2014).

Recently, researchers have paid more attention to roles of the plasma membrane in plant–microbe interactions (Jarsch and Ott, 2011). The plasma membrane of the plant cell, which is a cellular boundary to define individual cells, is actually continuous between cells as an integral component of plasmodesmata together with the ER that forms the desmotubule (the central structure of a plasmodesma) (Maule, 2008). In addition, the plasma membrane contains microdomains termed lipid rafts or membrane rafts, which are enriched in sterols and sphingolipids (Mongrand et al., 2004). The specialized microdomains on the plasma membrane are proposed to serve as platforms for the control of cellular processes including membrane fluidity, signal perception and transduction, macromolecular trafficking, and responses to pathogens (Mongrand et al., 2010). Remorins, plant-specific membrane proteins, are used as marker proteins of the membrane raft and localized on the plasma membrane and plasmodesmata (Raffaele et al., 2009a). A remorin family can be divided into six groups (Groups 1–6) (Raffaele et al., 2007). A recent study on remorins of *Solanaceae* species has suggested that one of Group 1 remorins of *Solanum tuberosum* (StREM1.3) may play an inhibitory role in the cell-to-cell movement of *Potato virus X* (PVX) in the genus *Potexvirus* (Raffaele et al., 2009a). StREM1.3 is shown to bind directly to the PVX MP (TGBp1) and impair the ability of TGBp1 and TMV MP to increase plasmodesmal permeability (Raffaele et al., 2009a; Perraki et al., 2012, 2014). The C-terminal region of StREM1.3 is responsible for self-oligomerization and targeting to the plasma membrane (Perraki et al., 2012; Gronnier et al., 2017). In contrast, the *Arabidopsis thaliana* remorins in Group 4 are reported to have a role as positive regulators for infections by geminiviruses such as *Beet curly top virus* and *Beet severe curly top virus* via interaction with the SNF-related kinase 1 (SnRK1) (Son et al., 2014). These results suggest that remorins in different groups may have different roles in controlling virus susceptibility depending on virus species.

Tobacco (*Nicotiana tabacum*) is a representative host for tobamoviruses. In a genome database of tobacco^1^, we can find multiple remorin genes including the previously reported NtREM1.2, which is categorized in Group 1 and closely related to StREM1.3 phylogenetically (Raffaele et al., 2009a). The *NtREM1.2* transcripts are expressed highly in various organs of tobacco (Raffaele et al., 2009b). In this study, we examined the relationship between a tobacco remorin and virus infection by using an *NtREM1.2* homologous cDNA isolated from Samsun NN tobacco, termed *Nt(sNN)REM1.2*. Transient expression experiments using fluorescently tagged proteins suggested that the subcellular localization of Nt(sNN)REM1.2 was altered by ToMV infection and the co-expression of the 126K protein or the MP, promoting the formation of discrete Nt(sNN)REM1.2 aggregates on the plasma membrane. The BiFC analysis demonstrated that Nt(sNN)REM1.2 interacted with itself and the MP in living cells. Our data also indicated that the transiently overexpressed Nt(sNN)REM1.2 in *N. benthamiana* exerted a promotive but not an inhibitory effect on the cell-to-cell movement of ToMV. Based on these results, we discuss possible biological roles of the tobacco remorin in tobamovirus infection.

## Materials and Methods

### Plants and Growth Conditions

*Nicotiana benthamiana* was grown on Rock Fiber blocks (Nittobo, Tokyo, Japan) at 25°C with a 16 h light/8 h dark photoperiod for 2 weeks. Seedlings on the Rock Fiber blocks were transferred to pots filled with vermiculite and grown under the same conditions, being fertilized with 0.1% (v/v) Hyponex solution (Hyponex Japan, Osaka, Japan) once a week. 7–9 week-old plants were used and kept at 25°C after bombardment and agroinfiltration until observation.

### Plasmid Construction

The plasmids described below were constructed using conventional or Gateway cloning techniques (Invitrogen, Carlsbad, CA, United States). Coding sequences in the plasmids amplified by polymerase chain reaction (PCR) were confirmed by sequencing.

For construction of pTH2-126K/183K-GFP, the 126K/183K-encoding sequence was firstly amplified by PCR using pTLW3 that is an expression plasmid with wild-type ToMV genomic cDNA (Hamamoto et al., 1993) and a primer set of 126KdTAG/NcoI/F1 (5′-GAACCATGGCATACACACAAACAGC-3′; the NcoI recognition sequence is underlined) and 183KdTAA/NcoI/R1 (5′-CACCCATGGCACAACTAGAGCCATCAAGAA-3′; the NcoI recognition sequence is underlined). The PCR product was digested with NcoI and ligated into an NcoI-cut large fragment obtained from the GFP-encoding plasmid, 35Ω-sGFP(S65T) (Chiu et al., 1996), generating pTH2-126K-GFP.

pART7-NtREM-YFP and pART7-NtREM-DsRed were constructed as follows. Total RNA was extracted from *N. tabacum* cv Samsun NN using the NucleoSpin RNA Plant Kit (Takara, Shiga, Japan). First strand cDNA was synthesized from the total RNA extract with an oligo-dT primer, and ReverTra Ace reverse transcriptase (Toyobo, Osaka, Japan). To obtain the NtREM cDNA without the termination codon, first PCR was carried out using the first strand cDNA as templates and a primer set of attB1/NtREM/F1 (5′-AAAAAGCAGGCTACCATGGCAGAAGTAGAAGTTAA-3′) and attB2/NtREM/R2 (5′-AGAAAGCTGGGTTAAAACATCCAAGGAGTTTCT-3′). Second, PCR was performed with purified PCR products and attB adaptor primers (Sasaki et al., 2009). The second PCR products were purified and introduced to the entry vector pDONR/Zeo (Invitrogen). The resulting entry plasmid (pDONR/Zeo-NtREMdTAA) was recombined with the destination vectors pART7-GWC-YFP and pART7-GWC-DsRed (Sasaki et al., 2009), generating pART7-NtREM-YFP and pART7-NtREM-DsRed, respectively.

pART7-DsRed-NtREM was constructed as follows. pART7 (Gleave, 1992) was digested with SmaI and ligated with the Gateway cassette A from the Gateway Conversion kit (Invitrogen). The resulting plasmid (pART7-GWA) was linearized by digestion with EcoRI and KpnI. The DsRed cDNA sequence was amplified by PCR using pART7-DsRed (Sasaki et al., 2009) and a primer set of DsRed/EcoRI/F2 (5′-AAAAAGAATTCATGGACAACACCGAGGACGT-3′; the EcoRI recognition sequence is underlined) and DsRed/KpnI/R (5′-AAAAAGGTACCCTGGGAGCCGGAGTGGCGGG-3′; the KpnI recognition sequence is underlined), digested with EcoRI and KpnI, and ligated into the linearized pART7-GWA, generating the destination vector pART7-DsRed-GWA. An entry plasmid pDONR/Zeo-NtREM that contains the NtREM cDNA with the termination codon was obtained in the same way as pDONR/Zeo-NtREMdTAA was made, except that attB2/NtREM/R1 (5′-AGAAAGCTGGGTTTAAAAACATCCAAGGAGTT-3′) was used for first PCR instead of attB2/NtREM/R2. pDONR/Zeo-NtREM was recombined with pART7-DsRed-GWA.

pART7-erGFP was constructed as follows. The erGFP-coding sequence was amplified by PCR using pGLW3-erGFP that is a binary plasmid for inoculation of ToMV-erGFP (Sasaki et al., 2013) and a primer set of HindIII/erGFP (5′-AAAAAAAGCTTATGAAGACTAATCTTTTTCT-3′; the HindIII recognition sequence is underlined) and XbaI/erGFP (5′-TTTTTTCTAGATTAAAGCTCATCATGTTTGT-3′; the XbaI recognition sequence is underlined). The PCR product was digested with HindIII and XbaI and ligated into a large fragment obtained by digestion of pART7 (Gleave, 1992) with HindIII and XbaI, generating pART7-erGFP.

pGWnV3-NtREM and pGWcV-NtREM were generated by recombination between pDONR/Zeo-NtREMdTAA and the destination vectors pGWnV3 and pGWcV, respectively (Nishimura et al., 2015).

pGWnV3-MP and pGWcV-MP were made by recombination of pDONR-ToMV30K (Haque et al., 2008) with the destination vectors pGWnV3 and pGWcV, respectively (Nishimura et al., 2015).

For construction of pGWnV3-126K and pGWcV-126K, first PCR was carried out by using pTH2-126K-GFP and primers, attB1/126kdTAG/F1 (5′-AAAAAGCAGGCTTAACCATGGCATACACACAAACA-3′) and attB2/126kdTAG/R1 (5′-AGAAAGCTGGGTTTTGAGTACCTGCATCTACTT-3′). Second PCR was performed with purified PCR products and attB adaptor primers (Sasaki et al., 2009). The second PCR products were purified and introduced to the entry vector pDONR/Zeo (Invitrogen) to generate pDONR/Zeo-126K. pGWnV3-126K and pGWcV-126K were made by recombination of pDONR/Zeo-126K with the destination vectors pGWnV3 and pGWcV, respectively (Nishimura et al., 2015).

pGreenII-NtREM-DsRed was constructed as follows. After pART7-NtREM-DsRed was digested with NotI, a fragment containing the NtREM-DsRed coding sequence was ligated to the fragment from the binary vector pGreenII 0000^2^ that was digested with NotI, generating pGreenII-NtREM-DsRed.

pGreenII-DsRed was made by digesting pGreenII-NtREM-DsRed with NcoI and self-ligated.

### Particle Bombardment- and Agrobacterium-Mediated Transient Gene Expression

Particle bombardment of *N. benthamiana* leaves with piL.erG3L (Sasaki et al., 2009), pTH2-126K-GFP (Sasaki et al., 2014), pART7-MP-YFP (Sasaki et al., 2009), and expression plasmids constructed in this study was carried out as described previously (Sasaki et al., 2009). Infiltration of *Rhizobium radiobacter* (formerly *Agrobacterium tumefaciens*) strain GV3101(pMP90) to *N. benthamiana* leaves was performed as described previously (Haque et al., 2008). After agrobacterium transformants carrying pGreenII-DsRed, pGreenII-NtREM-DsRed, or pGLW3-erGFP (Sasaki et al., 2013) were incubated until OD600 reached about 0.6, each of the agrobacterium transformants carrying pGreenII-DsRed or pGreenII-NtREM-DsRed was mixed with that carrying pGLW3-erGFP at a 10:1 ratio. The total OD600 for infiltration was then adjusted to 0.11 by addition of incubation buffer (10 mM MgCl_2_, 10 mM MES, and a150 μM acetosyringone).

### Staining of the Plasma Membrane or Callose

Leaves of *N. benthamiana* at 21 h post-bombardment were treated with 50 μM FM4-64 or 0.01% (w/v) aniline blue for staining the plasma membrane or callose, respectively. After 3 h of staining the plasma membrane or callose, the leaves were observed with confocal laser microscopy (LSM710NLO; Carl Zeiss, Jena, Germany).

### Imaging of Fluorescent Proteins

For subcellular localization and time-lapse analyses, the fluorescence of the target fluorescent proteins was observed at 24 h after bombardment with the LSM710NLO confocal laser microscope (Carl Zeiss). GFP/YFP and DsRed were excited with an argon laser (488 nm) and a diode-pumped solid state laser (561 nm), respectively, and detected by Gallium arsenide phosphide (GaAsP) detectors for each fluorescence.

Aniline blue was excited with a blue diode laser (405 nm) and its fluorescence was detected in the range between 410 and 528 nm. Images were captured in the sequential mode and processed with the software ZEN 2009 (Carl Zeiss).

For measurement of fluorescent infection sites of ToMV-erGFP, GFP was detected at 48 and 72 h after infiltration with an all-in-one fluorescence microscope (BZ-X700; Keyence, Osaka, Japan).

The areas of fluorescent regions were measured using the ImageJ software^3^.

### Immunological Analysis

Preparation of protein samples from agrobacterium-infiltrated or uninfiltrated (healthy) leaves, SDS-PAGE, and western blotting were performed as described previously (Sasaki et al., 2009). DsRed and NtREM-DsRed were detected by using anti-DsRed antibody (Living Colors A.v. Monoclonal Antibody JL-8) (Takara) and stabilized goat anti-mouse HRP-conjugated secondary antibody (Pierce, Thermo Fisher Scientific, Rockford, IL, United States).

## Results

### Cloning of *NtREM1.2* Homologous cDNA From Samsun Tobacco

**FIGURE 1:**
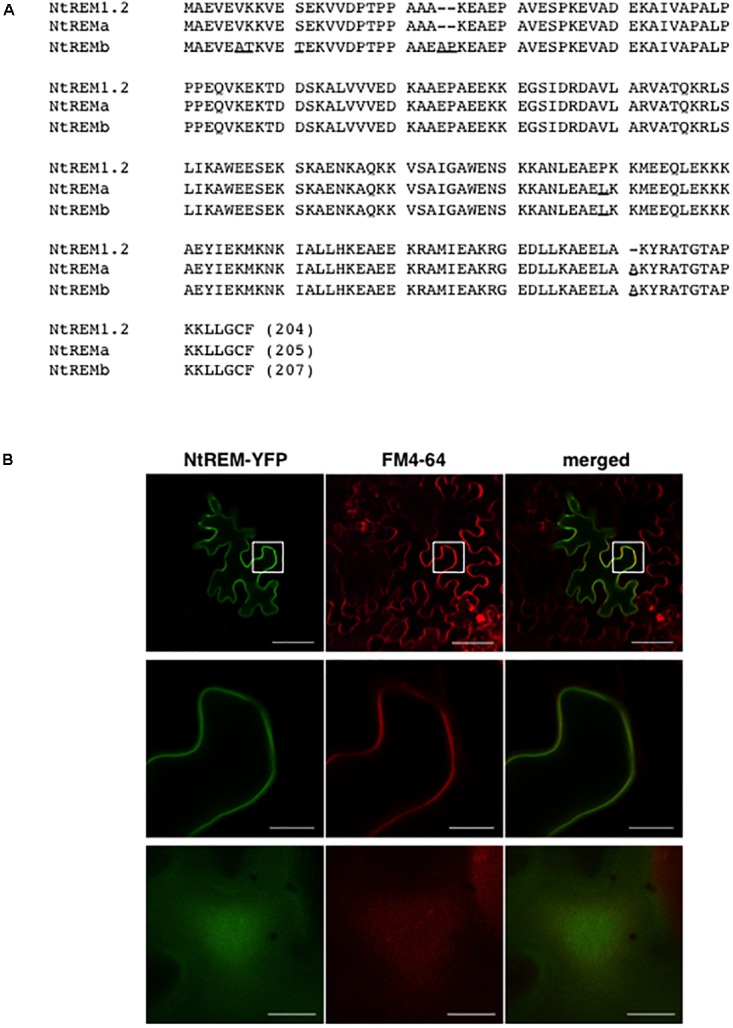
Amino acid sequence and plasma membrane localization of NtREM(sNN)1.2. **(A)** Amino acid sequences of NtREM(sNN)1.2 (=NtREMa) and NtREMb are aligned with that of NtREM1.2 (EB449751.1). Amino acids that are different from those of NtREM1.2 are underlined. Hyphens indicate gaps in amino acid sequences. **(B)** Leaves of *Nicotiana benthamiana* were bombarded with pART7-NtREM-YFP and stained with the FM4-64 solution at 21 h post-bombardment. The epidermal cells stained for 3 h were observed with confocal laser microscopy. Upper and lower panels show a transverse section of the epidermis and the peripheral surface of the bombarded cell, respectively. Middle panels show the magnified areas encompassed by squares in the upper panels. Bars in upper panels and middle and lower panels indicate 50 and 10 μm, respectively.

We attempted to isolate the cDNA of *NtREM1.2* (accession number, EB449751.1) from tobacco cv. Samsun NN. As a result, we obtained two cDNAs (termed *NtREMa* and *NtREMb*) with high identity to NtREM1.2. Comparison of the deduced amino acid sequences of NtREMa and NtREMb with NtREM1.2 (**Figure 1**) revealed 99.0 and 96.1% identity, respectively. Characteristically, NtREMb has two extra amino acids in the N terminal region, which are absent in NtREM1.2 and NtREMa. Through the BLAST search on the Sol Genomics Network database^4^, we found that two tobacco cultivars (TN90 and BX) and *N. tomentosiformis* (paternal parent of tobacco) had an ORF sequence with 100% identity of *NtREMa*. On the other hand, homologous ORF sequences with more than 98% but not 100% identity of *NtREMb* were found in three tobacco cultivars (TN90, BX and K326) and *N. sylvestris* (maternal parent of tobacco). All of the *NtREMb* homologs encode proteins with the above-mentioned two extra amino acids, these results suggested that NtREMa and NtREMb in tobacco, which should be orthologous between its parental species, were derived from *N. tomentosiformis* and *N. sylvestris*, respectively. *NtREMa* was selected for further analyses as the closest homolog of *NtREM1.2* and renamed *Nt(sNN)REM1.2*.

### Subcellular Localization of Fluorescently Labeled Nt(sNN)REM1.2 Proteins

In order to confirm the plasma membrane localization of Nt(sNN)REM1.2, we performed a bombardment assay using a cDNA clone of Nt(sNN)REM1.2 fused C-terminally with YFP (NtREM-YFP). Leaves of *N. benthamiana* were bombarded with the cDNA clone and observed under a confocal microscope at 24 h post-bombardment (hpb). Fluorescence of YFP was observed to distribute almost uniformly along the cell wall and the cell peripheral surface (**Figure 1**). Co-localization of NtREM-YFP with the plasma membrane marker FM4-64 (red fluorescence) indicated that Nt(sNN)REM1.2 is a plasma membrane-associated protein.

### Infection With ToMV-erGFP Promotes the Formation of NtREM-DsRed Aggregates

**FIGURE 2:**
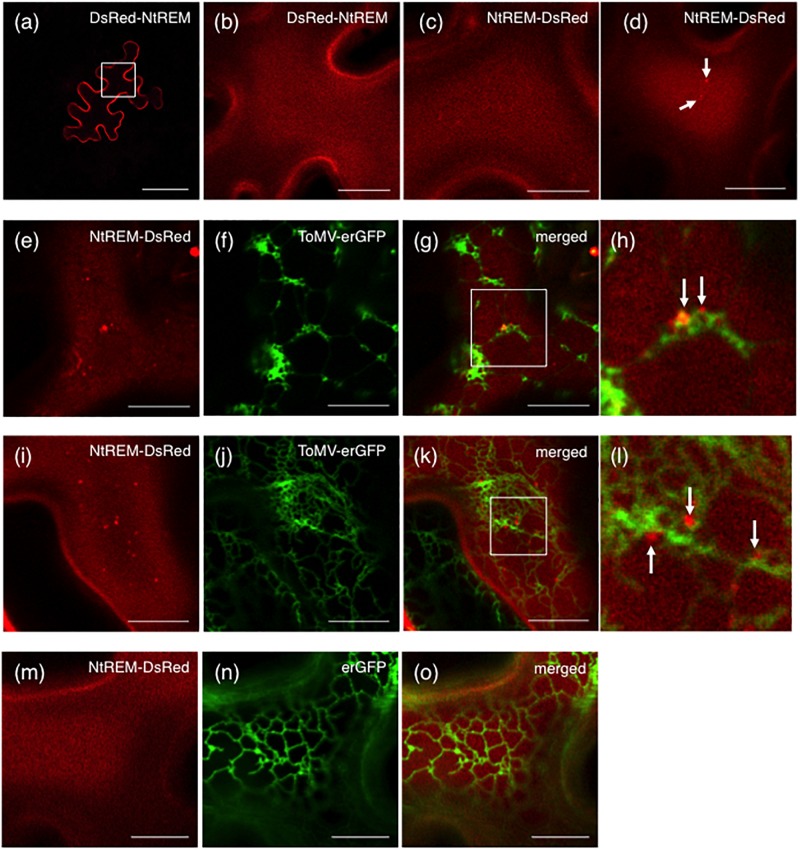
Infection with ToMV-erGFP promotes the formation of ER-associated NtREM-DsRed aggregates. Leaves of *N. benthamiana* were bombarded with pART7-DsRed-NtREM **(a,b)**, or pART7-NtREM-DsRed alone **(c,d)** or in combination with piL.erG3L **(e–l)** or pART7-erGFP **(m–o)**. Abaxial epidermal cells were observed at 24 **(a–h,m–o)** or 48 **(i–l)** h post-bombardment with confocal laser microscopy. DsRed-NtREM and NtREM-DsRed were observed similarly to localize on the plasma membrane in a transverse section **(a)** and surface layers **(b–d)** when expressed alone. Cells infected with ToMV-erGFP **(e–l)** but not those co-expressing erGFP **(m–o)** showed more aggregates of NtREM-DsRed along ER structures indicated by erGFP. The areas encompassed by squares in **(g,k)** are magnified in **(h,l)**, respectively. Arrows indicate aggregates of NtREM-DsRed. Bars in **(a)** and **(b–g,i–k,m–o)** indicate 50 and 10 μm, respectively.

**FIGURE 3:**
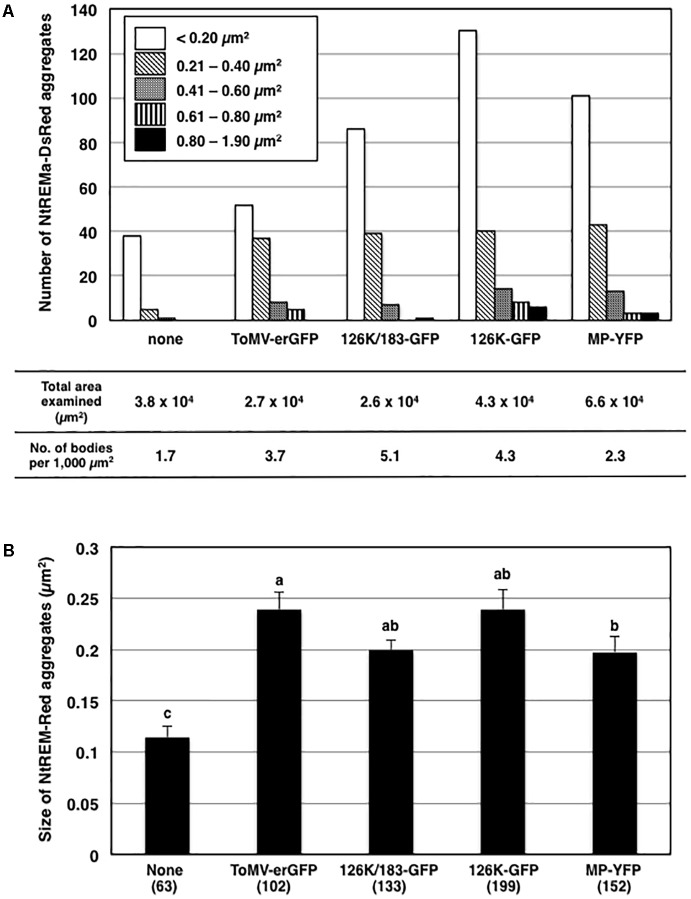
Effects of virus infection and transient expression of viral proteins on the formation of NtREM-DsRed aggregates. The numbers and the sizes of NtREM-DsRed aggregates in cells that were infected with ToMV-erGFP or co-expressed with each of 126K/183K-GFP, 126K-GFP, and MP-YFP were determined at 24 h post-bombardment. The numbers of NtREM-DsRed aggregates with indicated areas and per 1,000 μm^2^ (calculated based on the total areas of the fluorescent cell surface examined in at least three independent experiments) are shown in **(A)**. The sizes of NtREM-DsRed aggregates (μm^2^) are shown in **(B)**. Different letters (a, b, and c) above the bars indicate significant differences at *p* < 0.05 by the Steel-Dwass test. Values are the means ± SE of the size of aggregates in the total numbers of bombarded cells examined (in parentheses) in at least three independent experiments.

As observed for NtREM-YFP, bombardment with cDNA clones of Nt(sNN)REM1.2 fused with DsRed at the N-terminus (DsRed-NtREM) (**Figures 2a,b**) or the C-terminus (NtREM-DsRed) (**Figure 2c**) resulted in similar, uniform distribution of fluorescence on the plasma membrane. In order to assess a role of Nt(sNN)REM1.2 in virus infection, we examined the change in the subcellular localization pattern of NtREM-DsRed upon infection of ToMV-erGFP that encodes the ER-targeting GFP (erGFP) instead of CP (Sasaki et al., 2009). We bombarded *N. benthamiana* leaves with the cDNA clone of NtREM-DsRed alone or in combination with the infectious clone of ToMV-erGFP. In cells without virus infection, we found occasionally a few small aggregates of NtREM-DsRed (**Figure 2d**) as well as DsRed-NtREM (Supplementary Figure S1). The average number per 1,000 μm^2^ and size of NtREM-DsRed aggregates in the non-infected cells were 1.7 and 0.11 μm^2^, respectively (**Figure 3**). In contrast, cells infected with ToMV-erGFP showed a relatively dispersed distribution of NtREM-DsRed and more and larger aggregates of NtREM-DsRed on the plasma membrane. At 24 hpb (early-to-mid infection), cortical ER networks were disrupted and NtREM-DsRed aggregates were associated closely with the coalescent cortical ERs (**Figures 2e–h**). Cortical ER networks were recovered at 48 hpb (late infection), and these aggregates located in the proximity of tubular ER structures (**Figures 2i–l**). Detailed measurement indicated that both of the number and the size of NtREM-DsRed aggregates were increased by infection of ToMV-erGFP. The average number per 1,000 μm^2^ and size of NtREM-DsRed aggregates in the virus-infected cells were 3.7 and 0.24 μm^2^, respectively (**Figure 3**). In addition, we investigated a non-specific effect of erGFP on the subcellular localization of NtREM-DsRed by co-bombardment of the NtREM-DsRed and erGFP cDNAs. Cells co-expressing erGFP showed a similar localization pattern of NtREM-DsRed to that observed in the cells expressing the NtREM-DsRed alone (**Figures 2m–o**) (Supplementary Figure S2). These results suggested that the virus infection altered specifically the subcellular localization of NtREM-DsRed and promoted the aggregation of NtREM-DsRed at sites close to cortical ERs.

### Transient Expressions of Viral Replication and Movement Proteins Promote the Formation of NtREM-DsRed Aggregates

ToMV-erGFP encodes the 126K and 183K replication proteins and MP but not the CP. One or more of these viral proteins were likely to be involved in the altered subcellular localization of NtREM-DsRed in ToMV-infected cells. In order to examine this possibility, we expressed NtREM-DsRed together with fluorescently labeled viral proteins and observed fluorescence at 24 hpb. In this experiment, we constructed a 126K/183K-GFP cDNA clone for a dual expression of the 126K protein and a GFP-fused 183K protein (183K-GFP) that is translated via ribosomal read-through of the stop codon of the 126K gene, and used cDNA clones of a GFP-fused 126K protein (126K-GFP) and a YFP-fused MP (MP-YFP) (Sasaki et al., 2009, 2014).

**FIGURE 4:**
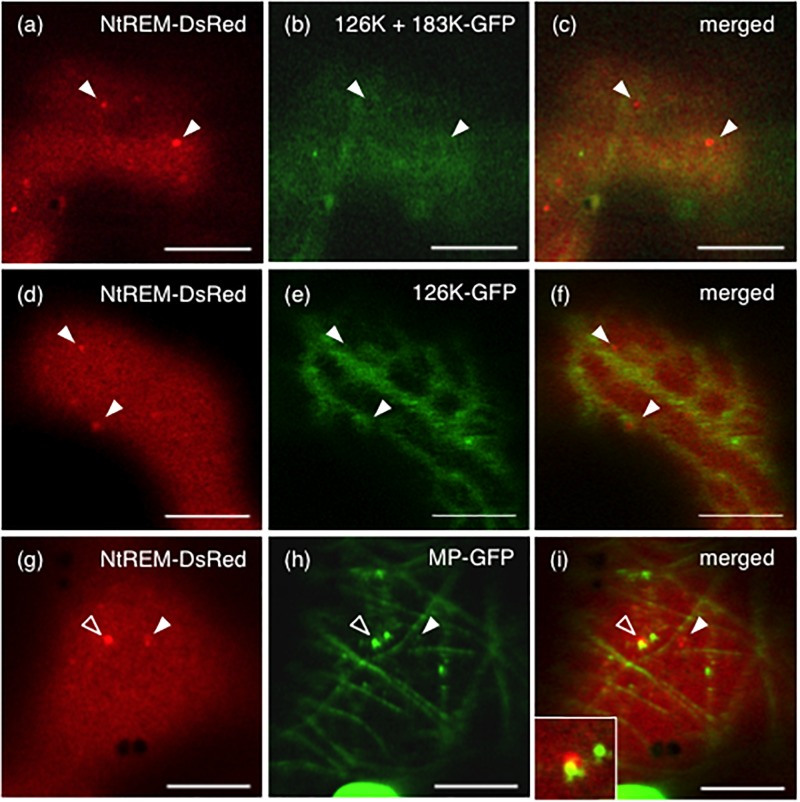
Formation of NtREM-DsRed aggregates in cells co-expressing the 183K-GFP and 126K, 126K-GFP, or movement protein-GFP. Leaves of *N. benthamiana* were bombarded with pART7-NtREM-DsRed in combination with pTH2-126K/183K-GFP **(a–c)**, pTH2-126K-GFP **(d–f)**, and pART7-MP-YFP **(g–i)** and observed at 24 h post-bombardment with confocal laser microscopy. Fluorescent images of NtREM-DsRed **(a,d,g)**, 183K-GFP **(b)**, 126K-GFP **(e)**, and MP-YFP **(h)** and their merged images **(c,f,i)** are presented. Note that the 126K protein and 183K-GFP are expressed in a cell bombarded with pTH2-126K/183K-GFP **(a–c)**. Open and closed arrowheads indicate the location of NtREM-DsRed aggregates. An inset in **(i)** shows a magnified region indicated by an open arrowhead. Bars indicate 10 μm.

**FIGURE 5:**
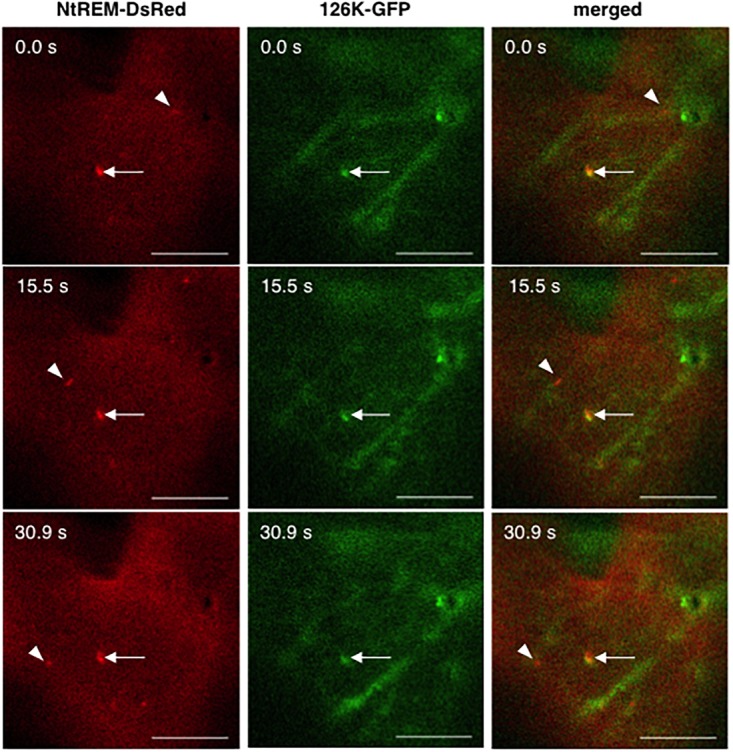
Dynamics of NtREM-DsRed aggregates with and without the association of 126K-GFP bodies. Leaves of *N. benthamiana* were bombarded with pART7-NtREM-DsRed in combination with pTH2-126K-GFP and observed at 24 h post-bombardment with confocal laser microscopy. Arrowheads indicate motile NtREM-DsRed aggregates while arrows show static NtREM-DsRed aggregates and 126K-GFP bodies, both of which located close to each other. Bars indicate 10 μm.

When NtREM-DsRed was co-expressed transiently with 126K and 183K-GFP (126K+183K-GFP) or 126K-GFP alone in epidermal cells, we found that the number and size of NtREM-DsRed aggregates increased similarly to the case of infection with ToMV-erGFP. The average number per 1,000 μm^2^ and size of the bodies were 5.1 and 0.20 μm^2^ for 183K-GFP, and 4.3 and 0.24 μm^2^ for 126K-GFP, respectively (**Figure 3**). There was no clear indication of an association of NtREM-DsRed aggregates with 183K-GFP that appeared to be distributed as uniformly as NtREM-DsRed (**Figures 4a–c**). Meanwhile, such NtREM-DsRed aggregates were shown to locate predominantly along the cytoplasmic distribution of 126K-GFP (**Figures 4d–f**). However, time-lapse imaging analysis showed that NtREM-DsRed aggregates associate with small 126K-GFP bodies were static while other NtREM-DsRed aggregates appeared to move with 126K-GFP transported by the cytoplasmic streaming (**Figure 5**). On the other hand, co-expression of MP-YFP also promoted the formation of larger NtREM-DsRed aggregates than those found in cells expressing NtREM-DsRed alone. The average number per 1,000 μm^2^ and size of the aggregates were 2.3 and 0.20 μm^2^ (**Figure 3**). As reported for TMV MP and StREM1.3 (Amari et al., 2014), there was no perfect co-localization of the MP and the remorin. However, NtREM-DsRed aggregates were sometimes found to be associated closely with filamentous and granular (body) structures of MP-YFP (**Figures 4g–i**). These collective results suggested that the overexpression of the replication proteins and MP triggered the formation of aggregates of Nt(sNN)REM1.2 on the plasma membrane.

### NtREM Interacts With ToMV MP *in Planta*

**FIGURE 6:**
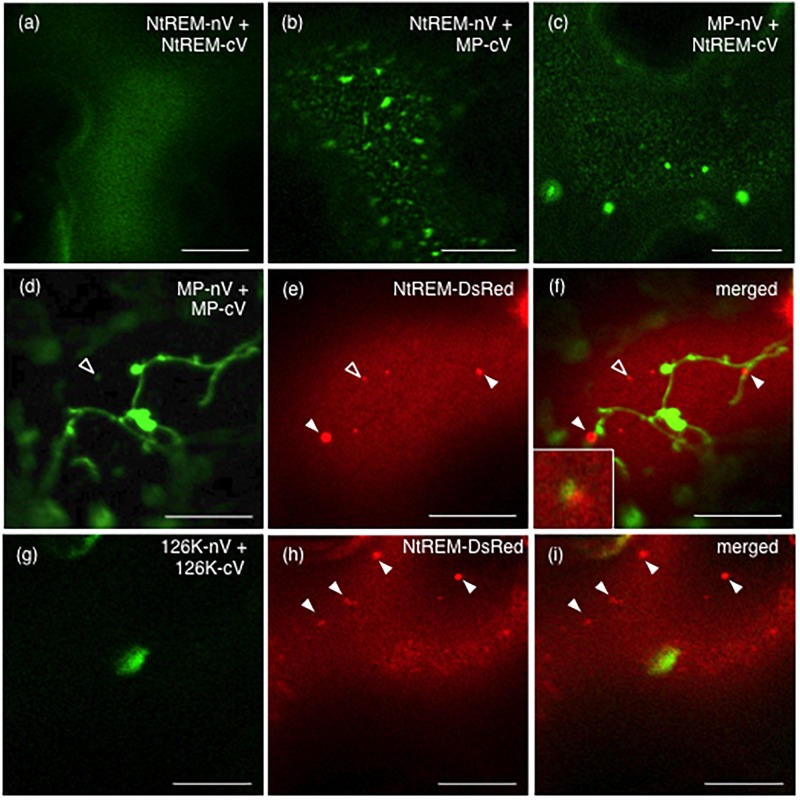
Nt(sNN)REM1.2 interacts with itself and the ToMV movement protein on the peripheral cell surface. BiFC experiments were performed by bombardment of *N. benthamiana* leaves with pGWnV3-NtREM and pGWcV-NtREM **(a)**, pGWnV3-NtREM and pGWcV-MP **(b)**, pGWnV3-MP and pGWcV-NtREM **(c)**, pGWnV3-MP, pGWcV-MP, and pART7-NtREM-DsRed **(d–f)**, or pGWnV3-126K, pGWcV-126K, and pART7-NtREM-DsRed **(g–i)**. Open and closed arrowheads indicate NtREM-DsRed aggregates. An inset in **(f)** shows a magnified region indicated by the open arrowhead. Fluorescence images were taken at 24 h post-bombardment with confocal laser microscopy. Bars indicate 10 μm.

In order to investigate interactions of NtREM with viral proteins, we adopt a recently developed BiFC (bimolecular fluorescence complementation) system that can avoid a self-interaction of the Venus fluorescent protein itself and detect specific protein interaction *in planta* (Nishimura et al., 2015). pGWnV3 and pGWcV-derived plasmids were used for expression of the N-terminal (nV) and C-terminal (cV) fragments of Venus fused to the C-terminal end of the target proteins, respectively. pGWnV3-NtREM, pGWcV-NtREM, pGWnV3-MP, and pGWcV-MP were used for the expression of NtREM-nV, NtREM-cV, MP-nV, and MP-cV, respectively. After the expression plasmids for BiFC were bombarded to *N. benthamiana* leaves, fluorescence of Venus was detected at 24 hpb. We observed the uniform distribution of fluorescence on the plasma membrane by co-expression of NtREM-nV and NtREM-cV. This demonstrated that Nt(sNN)REM1.2 interacts with itself (**Figure 6a**), consistent with previous reports for other remorins (Bariola et al., 2004; Perraki et al., 2012; Tóth et al., 2012). Cells co-expressing NtREM-nV and MP-cV exhibited patchy fluorescent spots (**Figure 6b**). Similarly, a reciprocal experiment using NtREM-cV and MP-nV demonstrated patchy distribution of fluorescent spots (**Figure 6c**). These results suggested strongly a direct interaction between Nt(sNN)REM1.2 and ToMV MP. On the other hand, similar to the case of the co-expression with MP-YFP, when NtREM-DsRed was co-expressed with a combination of MP-nV and MP-cV, we found that NtREM-DsRed aggregates were associated closely but not co-localized perfectly with filamentous and granular structures of self-interacting MPs on the peripheral surface (**Figures 6d–f**). Furthermore, we also examined the interaction of the 126K protein with Nt(sNN)REM1.2 as well as the 126K protein itself by using pGWnV3-126K and pGWcV-126K. We could rarely observe fluorescent, large amorphous structures in cells co-expressing 126K-nV with 126K-cV, while those structures were not associated with NtREM-DsRed aggregates (**Figures 6g–i**). On the other hand, no fluorescence was observed in the case of 126K-nV and NtREM-cV (data not shown). These observations suggested that the 126K protein might not interact directly with Nt(sNN)REM1.2. Through the BiFC assays, we noticed that the localization patterns of fluorescent MP seemed to be different between MP-YFP and BiFC-combinations. Since the reconstitution of the split Venus protein can stabilize BiFC complexes (Robida and Kerppola, 2009), it was likely that the BiFC-mediated subcellular localizations of MP might be altered depending on the interacting protein.

### Interaction Between Nt(sNN)REM1.2 and MP Around Plasmodesmata

**FIGURE 7:**
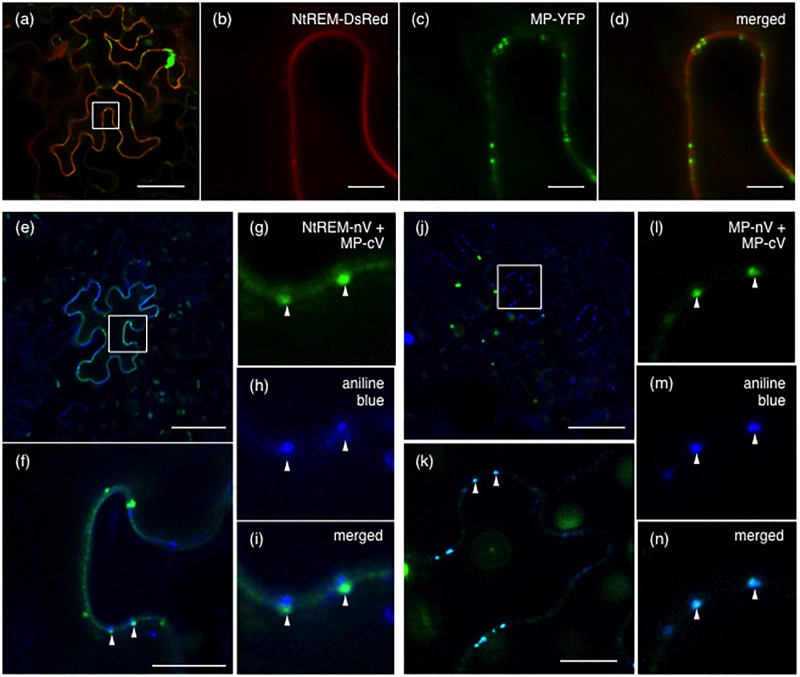
Interaction between NtREM and MP around plasmodesmata. Leaves of *N. benthamiana* were co-bombarded with pART7-NtREM-DsRed and pART7-MP-YFP **(a–d)**, pGWnV3-NtREM and pGWcV-MP **(e–i)**, or pGWnV3-MP and pGWcV-MP **(j–n)** and observed at 24 h post-bombardment with confocal laser microscopy. For detection of callose deposition at plasmodesmata, aniline blue solution was infiltrated to the bombarded leaves 3 h before observation. Areas encompassed by squares in **(a,e,j)** are magnified in **(b–d,f,k)**, respectively. Arrowheads in **(f,k)** correspond to those in **(g–i,l–n)**, respectively. Bars in **(a,e,j)**, **(b–d)**, and **(f,k)** indicate 50, 5, and 10 μm, respectively.

Since our BiFC analysis indicated that Nt(sNN)REM1.2 could interact directly with MP on the plasma membrane, we examined whether the two proteins are co-localized at plasmodesmata that are lined with the plasma membrane by observing *N. benthamiana* cells co-expressing NtREM-DsRed and MP-YFP at 24 hpb. Strong spots of MP-YFP, indicative of its plasmodesmal localization, were found on the layer of NtREM-DsRed in the transverse section of the cells (**Figures 7a–d**). However, there was little accumulation of NtREM-DsRed specifically on those spots. This observation was similar to the previous study showing that TMV MP and StREM1.3 are not co-localized substantially on the plasma membrane (Amari et al., 2014). Then, we further investigated the association of Nt(sNN)REM1.2 and MP with plasmodesmata by co-expressing NtREM-nV and MP-cV and staining the bombarded cells with aniline blue at 24 hpb to visualize callose deposition at plasmodesmata. The Venus fluorescence in the transverse section was detected mostly along the cell wall (**Figures 7e–i**), confirming the direct interaction between NtREM-nV and MP-cV on a wide area of the plasma membrane. In addition, we occasionally observed fluorescent spots of Venus, which were adjacent to but not co-localized with aniline blue-stained spots (**Figures 7g–i**). On the other hand, in control cells co-expressing MP-nV and MP-cV, fluorescent spots of Venus were co-localized perfectly with aniline blue-stained spots (**Figures 7j–n**). Thus, the results from the BiFC analysis suggested that Nt(sNN)REM1.2 and MP might interact at specific sites around plasmodesmata. Although we did not detect any clear accumulation of NtREM-DsRed together with MP-YFP either at or around plasmodesmata (**Figures 7a–d**), it was possible that BiFC-mediated stabilization of protein–protein interaction (Robida and Kerppola, 2009) might allow us to detect an innately temporary interaction between Nt(sNN)REM1.2 and MP around plasmodesmata.

### Transient Expression of NtREM-DsRed Has a Promotive Effect on Continuous Cell-to-Cell Movement of ToMV-erGFP

**FIGURE 8:**
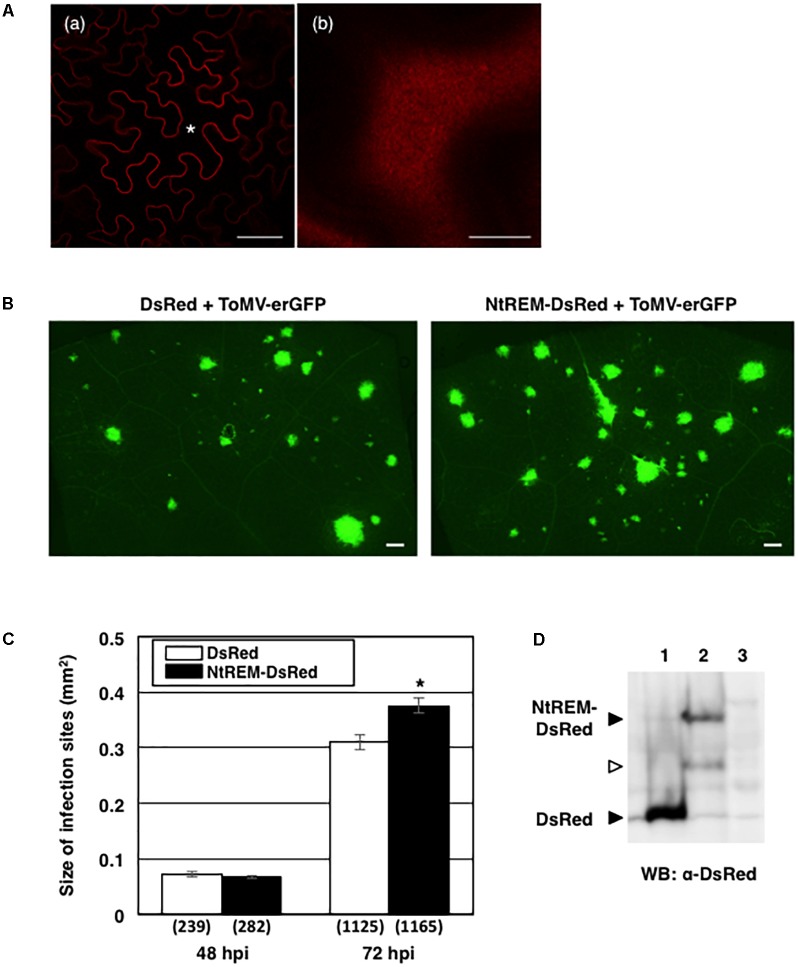
Agrobacterium-mediated transient expression of NtREM promotes ToMV cell-to-cell movement. Leaves of *N. benthamiana* were infiltrated with agrobacteria carrying pGLW3-erGFP (for inoculation of ToMV-erGFP) together with those carrying pGreenII-DsRed (for expression of DsRed) or pGreenII-NtREM-DsRed (for expression of NtREM-DsRed) and observed at 48 and 72 h post-infiltration (hpi) with an all-in-one fluorescence microscope. **(A)** Cells expressing NtREM-DsRed transiently after infiltration with agrobacteria carrying pGreenII-NtREM-DsRed together with those carrying pGLW3-erGFP were observed at 48 h post-infiltration with a confocal laser microscope. **(a,b)** Show a transverse section of the epidermis and a peripheral surface of the cell indicated by an asterisk, respectively. Bars in **(a,b)** indicate 50 and 10 μm, respectively. **(B)** Fluorescent images of infiltrated areas were taken at 72 hpi. **(C)** Average sizes of fluorescent infection sites were calculated based on the total number of infection sites in six different leaves (show in parenthesis). Error bars indicate SE of the average. An asterisk indicates a statistically significant difference by the Student’s *t-*test (*p* < 0.05). **(D)** Closed arrows indicate DsRed and NtREM-DsRed that were detected by anti-DsRed antibody in protein samples extracted from infiltrated (lanes 1 and 2) but not uninfiltrated tissues at 72 dpi (lane 3). An open arrow indicates possible degraded NtREM-DsRed.

We examined whether the transient overexpression of NtREM-DsRed by the above-mentioned bombardment method influenced the initial cell-to-cell movement of ToMV-erGFP in *N. benthamiana*. The percentage of multiple-cell infection sites at 48 hpb and the average number of cells in the multiple cell infection sites were comparable between bombarded tissues overexpressing DsRed and NtREM-DsRed transiently (Supplementary Figure S3), indicating that the overexpressed Nt(sNN)REM1.2 had no effect on the initial movement of the virus. Since the expression of NtREM-DsRed was restricted to bombarded cells only, we further investigated whether the transient overexpression of NtREM-DsRed in the whole leaf tissue by agroinfiltration could interfere with continuous cell-to-cell movement of the virus. In this analysis, *N. benthamiana* leaves were infiltrated with agrobacterium transformants for the expression of DsRed or NtREM-DsRed, together with that for inoculation of ToMV-erGFP. Confocal microscopic observation confirmed that agrobacterium-mediated expression of NtREM-DsRed also resulted in a uniform distribution on the plasma membrane (**Figure 8A**). Observation at 48 and 72 hours post-infiltration (hpi) with an all-in-one fluorescence microscope demonstrated that there was no significant increase in the size of fluorescent infection sites at 48 hpi, but there was a statistically significant difference at 72 hpi (**Figures 8B,C**) (Supplementary Figure S4). Western blot analysis demonstrated that both of the DsRed and NtREM-DsRed accumulated to detectable levels (**Figure 8D**). Collectively, our data suggested that transient overexpression of NtREM-DsRed had a promotive effect on continuous cell-to-cell movement of ToMV-erGFP in *N. benthamiana*.

## Discussion

In this study, our subcellular localization and BiFC analyses has demonstrated that Nt(sNN)REM1.2, a putative membrane raft-associated protein, is localized on the plasma membrane under normal conditions. Interestingly, irrespective of the type or the position of fluorescent proteins, the N- or C-terminally tagged Nt(sNN)REM1.2 fusions were detected uniformly throughout the plasma membrane plane. This localization pattern is inconsistent with the patchy distribution of the best-studied potato remorin, StREM1.3, which is also known as a typical membrane raft marker (Raffaele et al., 2009a). Our results may suggest that the association of Nt(sNN)REM1.2 with membrane rafts under normal conditions is not as strong as that of StREM1.3. Although Nt(sNN)REM1.2 and StREM1.3 are categorized in the same group (Raffaele et al., 2007, 2009a; Gronnier et al., 2017), we have found that their amino acid sequences in the N-terminal region are relatively variable whereas the central and C-terminal regions are highly conserved (Supplementary Figure S5A). In addition, two remorins of *N. benthamiana* (i.e., NbREM1.2 and NbREM1.3) have recently reported to show similar patchy distribution patterns in supplementary data (Gronnier et al., 2017). As the sequence data of NbREM1.2 and NbREM1.3 are unavailable, we analyzed the most similar remorin sequence to Nt(sNN)REM1.2 from the *N. benthamiana* cDNA database of the Sol Genomics Network^5^. Our result shows that the amino acid sequences of the N-terminal region of Nt(sNN)REM1.2 and the *N. benthamiana* homolog are also varied (Supplementary Figure S5B). According to recent studies (Perraki et al., 2012; Gronnier et al., 2017), the C-terminal domain of StREM1.3 is responsible for localization at nanodomains on the plasma membrane. Thus, the difference in the subcellular localization patterns between these remorins may be due to different functions of their N-terminal regions, or possibly different experimental conditions. Our BiFC assays also suggest the possibility that the N-terminal region of Nt(sNN)REM1.2 may mediate its clustering on the plasma membrane during ToMV infection through direct or indirect interactions with viral components such as 126K and MP. Further analysis of the N-terminal regions of Nt(sNN)REM1.2 and its related remorins may reveal important domains and amino acids that influence the distribution on the plasma membrane as well as the association with membrane rafts.

The uniform distribution of NtREM-DsRed on the plasma membrane allowed us to easily study the alteration of its localization pattern and the formation of NtREM-DsRed aggregates. Co-expression assays using NtREM-DsRed, ToMV-erGFP, and GFP/YFP-tagged virus proteins showed that the transient expression of any of the virus proteins examined (i.e., the combination of the 126K and 183K proteins, the 126K protein, and the MP) as well as the virus infection can disturb the uniform localization of NtREM-DsRed and promote the formation of NtREM-DsRed aggregates. Such aggregation of NtREM-DsRed suggests that Nt(sNN)REM1.2-associated membrane rafts, which are arranged uniformly on the plasma membrane under normal conditions, may be redistributed and clustered in places where the virus replication protein(s) and/or the MP may accumulate to certain levels to form VRCs. Our observation of NtREM-DsRed aggregates in the proximity of tubular ER structures in cells infected by ToMV-erGFP is consistent with the current model in which VRCs containing the replication proteins and the MP are formed at ER/microtubule junctions that are linked to the plasma membrane (Amari et al., 2014; Heinlein, 2015). To support the idea, NtREM-DsRed aggregates also appear to be associated closely with small 126K-GFP and MP bodies that are associated with the ER/actin network (Reichel and Beachy, 1998; Liu et al., 2005; Sasaki et al., 2014) and filamentous structures of MP-YFP that are associated with microtubules (Heinlein et al., 1998; Más and Beachy, 1999; Ashby et al., 2006; Brandner et al., 2008). Thus, it is possible that Nt(sNN)REM1.2-containing membrane rafts may function as platforms that connect VRCs formed at ER/microtubule junctions to the plasma membrane and plasmodesmata.

Our BiFC assays have demonstrated that Nt(sNN)REM1.2 interacts with itself as reported for other remorins (Bariola et al., 2004; Perraki et al., 2012; Tóth et al., 2012) and that the self-interacting Nt(sNN)REM1.2 also distributed as uniformly on the plasma membrane as NtREM-DsRed. We also show that NtREM interacts directly with MP on the plasma membrane, from which MP is shown to be fractionated abundantly (Moore et al., 1992). Interestingly, the Nt(sNN)REM1.2-MP interaction caused patchy fluorescent spots in contrast to the uniform distribution of the fluorescence caused by the self-interaction of Nt(sNN)REM1.2. There results imply that the interaction between the two proteins on the membrane raft may occur at specific sites on the plasma membrane possibly with assistance of other yet undetermined host factors at ER/microtubule junction, and result in clustering membrane rafts. The arrangement of the fluorescent spots of the Nt(sNN)REM1.2-MP complex resembles a beads-on-a-string localization pattern of TMV MP, which is observed on the cell surface of protoplasts infected with a TMV mutant encoding GFP-fused MP (Heinlein et al., 1998). Furthermore, our close observation of the transverse cell sections has suggested that Nt(sNN)REM1.2-MP complexes may be formed at sites that are adjacent to, but distinct from, plasmodesmata where the self-interaction of the MP occurs. Thus, Nt(sNN)REM1.2 may play an important role in targeting ToMV MP from the plasma membrane to plasmodesmata. A recent study on the Arabidopsis synaptotagmin SYTA proposed that the plasma membrane protein functions in forming the contact site between the cortical ER and the plasma membrane, targeting the MP of another tobamovirus, *Turnip vein clearing virus* (TVCV), to plasmodesmata, and remodeling the contact sites to create virus replication sites for movement at plasmodesmata (Levy et al., 2015). It will be interesting to examine the relationship between remorins and synaptotagmins in tobacco in controlling the localization and movement function of tobamovirus MPs.

At present, we have no evidence that the 126K and 183K proteins interact directly with Nt(sNN)REM1.2, while at least the transient expression of the 126K protein is enough to induce the aggregation of NtREM-DsRed. It is likely that the mechanism of 126K to form such aggregates is different from the above-mentioned MP-mediated mechanism involving the direct interaction with Nt(sNN)REM1.2. Considering that some of NtREM-DsRed aggregates were observed to locate closely to 126K-GFP that is assumed to be associated with ER membranes (Heinlein, 2015), it is possible that ToMV replication protein(s) may be involved in rearranging and clustering Nt(sNN)REM1.2-containing membrane rafts indirectly by remodeling ER-plasma membrane contact sites to form VRC for virus replication and subsequently delivery of the virus genome to plasmodesmata.

Our result has demonstrated that the overexpression of NtREM-DsRed has no influence on the initial movement but can facilitate the continuous spread of ToMV-erGFP from the initially infected cell in *N. benthamiana*. This result suggests that Nt(sNN)REM1.2 may function as a positive regulator for ToMV spread after the initial movement. In the case of the closely related TMV, the initial movement is shown to be much slower than the subsequent movement in which the virus is proposed to spread between cells efficiently in the form of entire VRCs (Kawakami et al., 2004). Possibly, an effect of the overexpression of NtREM-DsRed on the initial movement of ToMV-erGFP was not apparent because the movement-promoting effect of the remorin could be masked by those of inherent remorins of *N. benthamiana* or specifically advantageous to the VRC movement stage. On the other hand, our result is contradictory to the recent reports that the overexpression of StREM1.3, NbREM1.2, and NbREM1.3 interferes with the cell-to-cell movement of PVX (Raffaele et al., 2009a; Gronnier et al., 2017). This different effect of Nt(sNN)REM1.2 on virus movement function from those of the other remorins may be explained by their variable N-regions (see above), or possibly due to different viruses tested. In addition, it should also be noted that the overexpressed StREM1.3 is shown to hamper plasmodesmal gating function of the MPs of not only PVX but also TMV (Perraki et al., 2014). Thus, it is important to determine whether Nt(sNN)REM1.2 prevents TMV MP from increasing the plasmodesmal permeability. Furthermore, although remorins in tobacco as well as tomato are immunologically detected at plasmodesmata (Raffaele et al., 2009a), the plasmodesmal localization of Nt(sNN)REM1.2 is unclear in the current study. Thus, it remains to be elucidated in future studies how Nt(sNN)REM1.2 is involved in the gating and transport functions of plasmodesmata.

In summary, our data presented here have suggested the involvement of the putative membrane raft-associated protein Nt(sNN)REM1.2 in ToMV infection via direct or indirect interaction with virus replication and MPs. Very recently, the tobamovirus resistance factor Tm-2^2^ of tomato is reported to interact with TMV MP at the plasma membrane but not plasmodesmata (Chen et al., 2017). This report validates the idea that the plasma membrane, and possibly the membrane raft, is a crucial site for MP to interact with not only susceptible host factors but also antiviral resistance factors. Further analyses of the interaction between Nt(sNN)REM1.2 and the virus proteins and identification of host factors that interact with Nt(sNN)REM1.2 on the membrane raft will help us understand the mechanism of intra- and intercellular movement of tobamoviruses and other plant viruses.HN and NS designed all the experiments in this study. ET and NS performed the experiments. All authors participated in writing this manuscript.

## Conflict of Interest Statement

The authors declare that the research was conducted in the absence of any commercial or financial relationships that could be construed as a potential conflict of interest.

## Footnotes

1 https://solgenomics.net/

2 https://www.addgene.org/vector-database/2982/

3 http://rsb.info.nih.gov/ij/

4 https://solgenomics.net

5 https://solgenomics.net/
