# Supplementary figures and images for: Altered Subcellular Localization of a Tobacco Membrane Raft-Associated Remorin Protein by Tobamovirus Infection and Transient Expression of Viral Replication and Movement Proteins
Source: Front Plant Sci. 2018 May 15;9:619. doi: 10.3389/fpls.2018.00619 (PMC5962775; doi:10.3389/fpls.2018.00619)

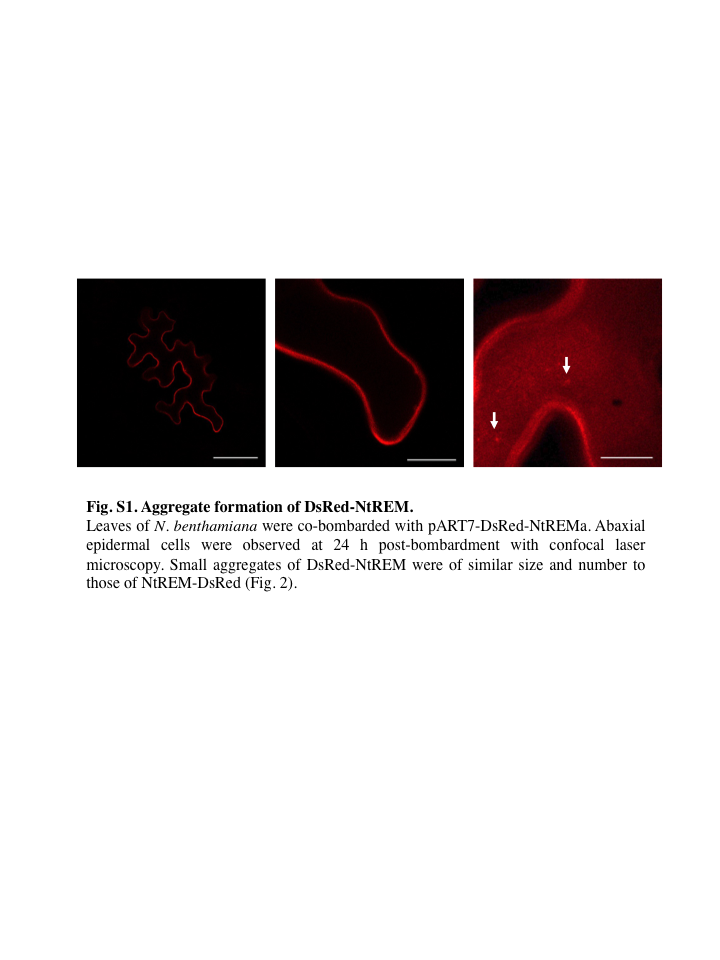

Supplement: Supplementary file 1 [file Image_1.TIFF]

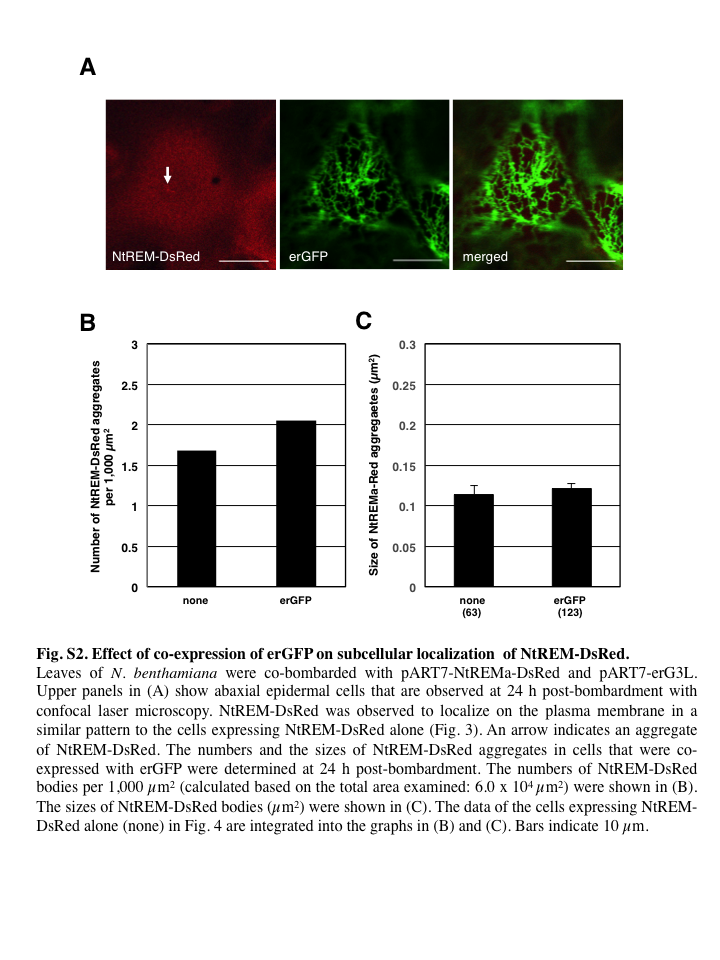

Supplement: Supplementary file 2 [file Image_2.TIFF]

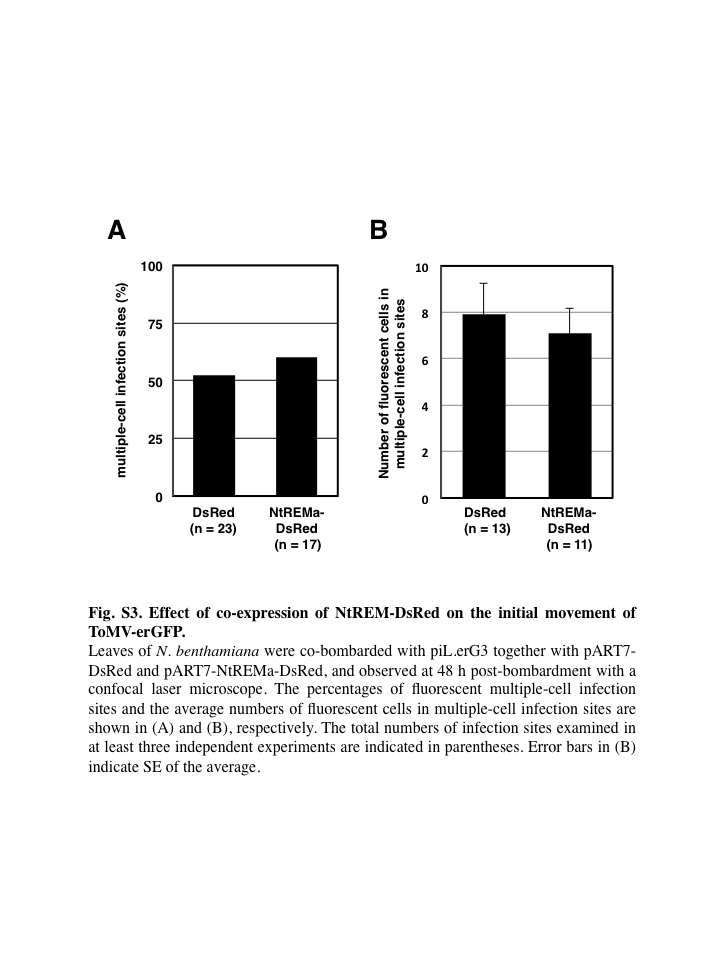

Supplement: Supplementary file 3 [file Image_3.TIFF]

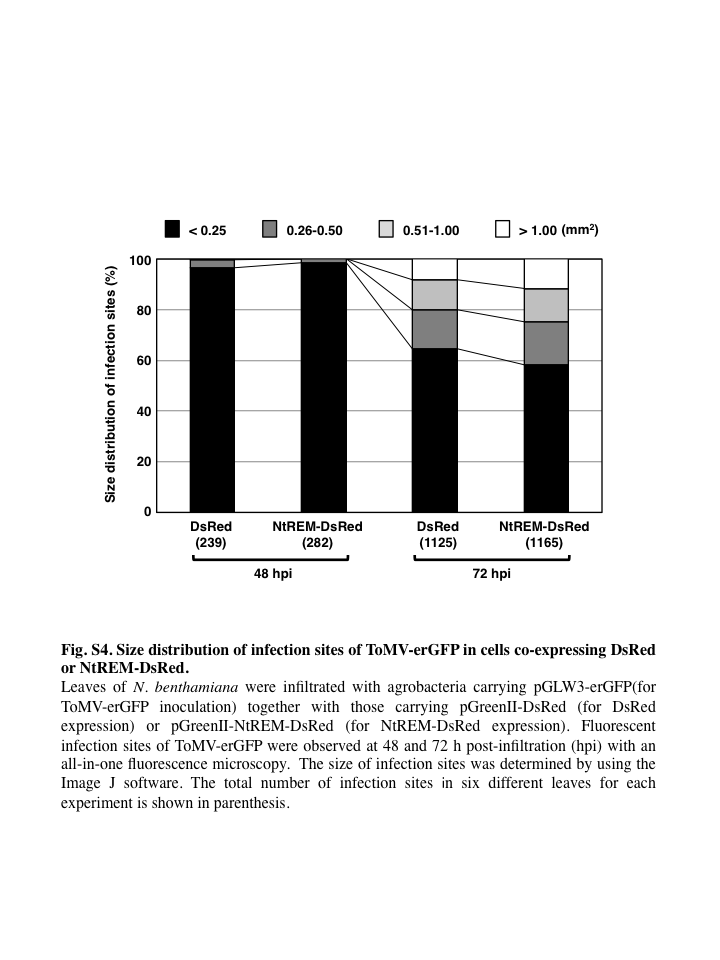

Supplement: Supplementary file 4 [file Image_4.TIFF]

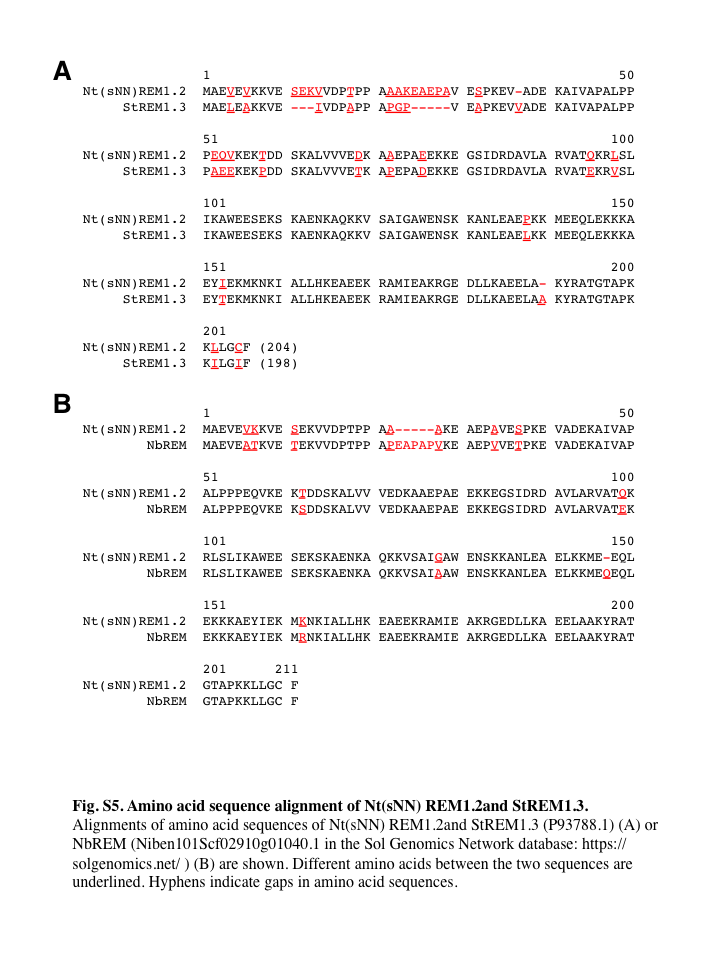

Supplement: Supplementary file 5 [file Image_5.TIFF]
